# Bemarituzumab plus mFOLFOX6 as first-line treatment in East Asian patients with FGFR2b-overexpressing locally advanced or metastatic gastric/gastroesophageal junction cancer: subgroup of FIGHT final analysis

**DOI:** 10.1007/s10120-024-01516-3

**Published:** 2024-06-11

**Authors:** Yoon-Koo Kang, Shukui Qin, Keun-Wook Lee, Sang Cheul Oh, In-Ho Kim, Jong Gwang Kim, Yong Li, Zhuchen Yan, Jin Li, Li-Yuan Bai, Catherine Chan, Akeem Yusuf, Anita Zahlten-Kümeli, Kate Taylor, Kensei Yamaguchi

**Affiliations:** 1 Department of Oncology Asan Medical Centre, University of Ulsan College of Medicine, 88, Olympic-Ro 43-Gil, Songpa-Gu, Seoul, 05505 South Korea; 2 Nanjing Tianyinshan Hospital, The First Affiliated Hospital of China Pharmaceutical University, Nanjing, China; 3 Seoul National University College of Medicine, Seoul National University Bundang Hospital, Seongnam, Gyeonggi-Do South Korea; 4 Department of Internal Medicine, Korea University Guro Hospital, Seoul, South Korea; 5 Department of Oncology, Seoul St. Mary’s Hospital, The Catholic University of Korea, Seoul, South Korea; 6 Kyungpook National University Chilgok Hospital, Daegu, South Korea; 7 The Fourth Hospital of Hebei Medical University, Shijiazhuang, Hebei China; 8 Tianjin Medical University Cancer Institute and Hospital, Tianjin, China; 9 Department of Oncology, Shanghai East Hospital, Shanghai, China; 10 Division of Hematology and Oncology, China Medical University Hospital, and China Medical University, Taichung, Taiwan; 11 Amgen Inc., Thousand Oaks, USA; 12 Amgen Ltd, Uxbridge, UK; 13 Gastroenterological Chemotherapy Department, The Cancer Institute Hospital of JFCR, Koto-Ku, Tokyo, Japan

**Keywords:** Bemarituzumab, FGFR2b, Gastric cancer, mFOLFOX6, Targeted therapy

## Abstract

**Background:**

In the FIGHT study (NCT03694522) bemarituzumab, a humanized monoclonal antibody selective for fibroblast growth factor receptor 2b (FGFR2b), plus mFOLFOX6 showed clinically meaningful efficacy in patients with FGFR2b-positive (2+/3+ membranous staining by immunohistochemistry) locally advanced unresectable/metastatic gastric/gastroesophageal cancer (G/GEJC). A meaningful proportion of patients in FIGHT were enrolled in East Asia, reflecting global epidemiology of G/GEJC.

**Methods:**

This subgroup analysis of the global, phase 2, double-blind FIGHT study included all patients enrolled in East Asian sites. Patients were randomized 1:1 to bemarituzumab-mFOLFOX6 (15 mg/kg and one 7.5 mg/kg dose on cycle 1, day 8) or matching placebo-mFOLFOX6. The primary endpoint was investigator-assessed progression-free survival (PFS). Secondary endpoints included overall survival (OS), objective response rate, and safety. Efficacy was evaluated after a minimum follow-up of 24 months.

**Results:**

The East Asian subgroup comprised 89 patients (57% of overall study population); 45 were randomized to bemarituzumab-mFOLFOX6 and 44 to placebo-mFOLFOX6. Median PFS (95% confidence interval [CI]) was 12.9 months (8.8–17.9) with bemarituzumab-mFOLFOX6 and 8.2 months (5.6–10.3) with placebo-mFOLFOX6 (HR 0.50, 95% CI 0.29–0.87); median OS (95% CI) was 24.7 months (13.8–33.1) vs 12.9 months (9.3–21.4), respectively (HR 0.56, 95% CI 0.32–0.96). Treatment benefit was more pronounced in patients with FGFR2b-positive G/GEJC in ≥ 10% of tumor cells. No new safety signals were reported.

**Conclusion:**

In East Asian patients with FGFR2b-positive advanced/metastatic G/GEJC enrolled in the global FIGHT study, bemarituzumab-mFOLFOX6 showed clinically meaningful outcomes over placebo-mFOLFOX6.

**Supplementary Information:**

The online version contains supplementary material available at 10.1007/s10120-024-01516-3.

## Introduction

Gastric/gastroesophageal junction cancer (G/GEJC) has a higher prevalence in East Asia compared with the rest of the world. Approximately 60% of G/GEJC cases and 58% of G/GEJC deaths in 2018 occurred in East Asia [1, 2]. Moreover, the age-standardized rate per 100,000 people was 22.36 cases and 15.94 deaths in East Asia, compared with 5.80 and 3.40, respectively, in Western Europe and 4.12 and 1.80, respectively, in North America [1, 2]. The incidence and mortality rates of G/GEJC have been successfully reduced in the past few decades across multiple East Asian countries/regions owing to screening accompanied by early treatment, *H. pylori* eradication, and economic development [3]. Despite the decline in incidence and the reduction in mortality rates for G/GEJC in Asia, 5-year survival rates remain quite poor [3]. Targeted therapies and immune checkpoint inhibitors have shown promising results in advanced G/GEJC, including in analyses/trials focusing on Asian patients [4]. Targeted and immune checkpoint inhibitors are increasingly available in Asia and have been incorporated into regional and national treatment guidelines [5–7]. As novel biomarkers emerge, they may reveal more opportunities to improve outcomes in locally advanced unresectable or metastatic G/GEJC via targeted therapeutics.

The IIb splice isoform of the fibroblast growth factor receptor 2 (FGFR2b) has emerged as a target for novel therapeutics in advanced G/GEJC [8]. The incidence of FGFR2b overexpression in G/GEJC has been estimated at 20% to 30% of cases, depending on the cutoff point for immunohistochemistry (IHC) staining [9–13]. The prognostic relevance of FGFR2b overexpression is not fully established, but some studies suggest it has been associated with poor prognosis [9–13]. Targeting FGFR2b in G/GEJC, therefore, represents a promising approach to improve survival outcomes in this patient population.

Bemarituzumab is a first-in-class, humanized, IgG1 monoclonal antibody that selectively inhibits FGFR2b signaling and enhances antibody-dependent cellular cytotoxicity against tumor cells expressing FGFR2b [14]. The phase 2, randomized, double-blind, global FIGHT trial (NCT03694522) evaluated bemarituzumab plus modified 5-fluorouracil, leucovorin, and oxaliplatin (mFOLFOX6) vs placebo plus mFOLFOX6 in patients with FGFR2b-overexpressing advanced G/GEJC [13, 15]. In the final analysis of the FIGHT study, bemarituzumab plus mFOLFOX6 extended median progression-free survival (PFS) and median OS with an acceptable safety profile [15]. Median PFS (median follow-up time, 6.8 months) was 9.5 months (95% CI 7.3–13.7) with bemarituzumab plus mFOLFOX6 and 7.4 months (95% CI 5.7–8.4) with placebo plus mFOLFOX6 (HR 0.72; 95% CI 0.49–1.08). Median OS was 19.2 months (95% CI 13.6–24.2) and 13.5 months (95% CI 9.3–15.9), respectively (HR, 0.77; 95% CI, 0.52–1.14) [19]. Most patients enrolled in FIGHT (63%) had FGFR2b overexpression in ≥ 10% of tumor cells as assessed by immunohistochemistry (IHC), defined as moderate (2+) to strong (3+) tumor staining intensity (FGFR2b ≥ 10% subgroup). In the FGFR2b ≥ 10% subgroup, median PFS (HR 0.43 in final analysis) and median OS (HR 0.52) were more pronounced than in the overall population [15].

FIGHT was a global randomized phase 2 trial with the majority of patients (57%) enrolled in East Asian sites. Here, we present the FIGHT final analysis results from all patients enrolled in East Asian sites.

## Methods

The trial design, protocol and primary results of the FIGHT study have been reported previously [13, 15]. Briefly, the phase 2 portion of FIGHT was a randomized, double-blind, placebo plus controlled study conducted at 164 sites across 18 countries. Of these, 31 sites were in East Asia (South Korea, China, Japan, and Taiwan).

The FIGHT global study evaluated the efficacy and safety of bemarituzumab plus mFOLFOX6 in patients with advanced G/GEJC not known to be HER-2 positive, with FGFR2b overexpression as assessed by IHC and/or *FGFR2* gene amplification as assessed by circulating tumor DNA (ctDNA) assay. Patients were randomized 1:1 to bemarituzumab plus mFOLFOX6 or placebo plus mFOLFOX6, and treatment continued through disease progression (Response Evaluation Criteria in Solid Tumors version 1.1 [RECIST v1.1]) or unacceptable toxicity. During the long-term follow-up period, patients were contacted every 3 (± 1) months for 24 months after the last patient was enrolled, or until death, loss to follow-up, consent withdrawal, or study termination, whichever occurred first. Data cutoff for this final analysis was May 13, 2022.

All patients provided written informed consent, and study protocols were approved by institutional ethics committees/institutional review boards at all sites.

### Population

Eligible patients were ≥ 18 years of age, had received no prior therapy for histologically confirmed advanced or metastatic G/GEJC, not known to be HER2-positive, had evaluable disease (RECIST version 1.1), had Eastern Cooperative Oncology Group (ECOG) performance status 0 or 1, and FGFR2b overexpression by IHC and/or *FGFR2* gene amplification by ctDNA assay. Patients who had received maximum of one dose of mFOLFOX6 during screening were eligible. FGFR2b overexpression by IHC was defined as any tumor cells with moderate (2+) to strong (3+) staining intensity, and cutoff for the ctDNA assay was 1.5X.

Exclusion criteria included untreated/symptomatic central nervous system metastasis, grade ≥ 2 common terminology criteria for adverse events (CTCAE) peripheral sensory neuropathy, acute or unstable corneal abnormalities (including but not limited to those that may increase the risk of developing a corneal ulcer as well as use of contact lenses).

### Treatment and randomization

Bemarituzumab dosing was 15 mg/kg every 2 weeks and one additional 7.5 mg/kg dose on cycle 1 day 8. The corresponding placebo treatment was administered with the same schedule as for bemarituzumab. The standard mFOLFOX6 regimen (oxaliplatin 85 mg/m^2^, leucovorin 400 mg/m^2^, and 5-fluorouracil 400 mg/m^2^ bolus followed by 2400 mg/m^2^ over approximately 48 h) was administered intravenously every 2 weeks in both arms. Randomization was stratified by geographic region (non-Asia [United States, European Union, United Kingdom, and Türkiye] vs China vs rest of Asia), prior treatment status (de novo vs adjuvant/neoadjuvant), and administration of a single dose of mFOLFOX6 during screening (yes vs no). Randomization was done using a permutated block scheme with a block size of four.

### Endpoints and assessments

The primary endpoint was PFS, and secondary endpoints included OS, objective response rate (ORR) (complete response [CR] or partial response [PR] according to investigator assessment of tumor lesions per RECIST v1.1), and incidence of adverse events. Duration of response (DOR) was an exploratory endpoint among patients who experienced a CR or PR according to RECIST v1.1. Adverse events were coded using the Medical Dictionary for Regulatory Activities version 25.0 and were graded using CTCAE version 5.0.

### Statistical analysis

Efficacy was analyzed in an intention-to-treat (ITT) population that included all randomized patients. Safety was analyzed in the safety analysis set that included patients who had received at least one dose of assigned treatment. The Kaplan–Meier method was used to estimate the median PFS and median OS and the associated 95% CIs in each treatment arm. HRs and 95% CIs were calculated using a stratified Cox regression model. Analysis of outcomes from the FGFR2b ≥ 10% overexpression subgroup (≥ 10% of tumor cells with moderate to strong staining as assessed by IHC) was prespecified in the overall population. Efficacy results in the East Asian subgroup are presented for the ITT population and among patients with FGFR2b ≥ 10% overexpression using the same statistical methodology. Data on adverse events are presented descriptively by number of patients and frequency in the safety population of the East Asian subgroup.

## Results

### Patient disposition and characteristics

Of the 473 patients prescreened at East Asian sites for FGFR2b overexpression, 45 were randomized to bemarituzumab plus mFOLFOX6 and 44 to placebo plus mFOLFOX6 (ITT; *N* = 89). The safety population included 44 patients in each treatment arm (ie, those who received at least one dose of the study treatment).

All patients had discontinued the study by the data cutoff date for the final analysis. Radiographic disease progression (*n* = 11 [24%] bemarituzumab plus mFOLFOX6, *n* = 25 [57%] placebo plus mFOLFOX6) and treatment-emergent adverse event (TEAE) (*n* = 21 [47%], *n* = 3 [7%], respectively) were the primary reasons for bemarituzumab/placebo treatment discontinuation. Death (*n* = 25 [57%] bemarituzumab plus mFOLFOX6, *n* = 32 [73%] placebo plus mFOLFOX6) was the primary reason for study discontinuation. Disease progression (*n* = 17 [38%] bemarituzumab plus mFOLFOX6, *n* = 27 [61%] placebo plus mFOLFOX6) was most common reason for death.

Baseline demographic and clinical characteristics were balanced between the treatment arms (Table 1). Across both treatment arms, the median age was 57 years and 63 (71%) were male. A total of 54 (61%) were enrolled in South Korea, 27 (30%) in China, 7 (8%) in Japan, and 1 (1%) in Taiwan. Gastric adenocarcinoma was the primary clinical diagnosis of cancer in 85 (96%) of the patients; ECOG status was 0 in 24 (27%) and 1 in 65 (73%); and 92% (*n* = 82) had disease stage IV at screening. One-third of patients received a single dose of mFOLFOX6 during screening (33% and 30% in the bemarituzumab plus mFOLFOX6 and placebo plus mFOLFOX6 arms, respectively). The median duration of exposure to study drug was 25.9 weeks (range, 2.0–94.6) in the bemarituzumab plus mFOLFOX6 arm and 27.4 weeks (range, 3.0–130.7) in the placebo plus mFOLFOX6 arm. The FGFR2b ≥ 10% subgroup comprised 29 (64%) patients from the bemarituzumab plus mFOLFOX6 arm and 31 (70%) patients from the placebo plus mFOLFOX6 arm.

**Table 1:** Patient demographics and characteristics in the FIGHT East Asian subgroup

	Bemarituzumab plus mFOLFOX6 (*N* = 45)	Placebo plus mFOLFOX6 (*N* = 44)
Age		
Median (range), years	57 (23–77)	58 (34–79)
≥ 65 years	12 (26.7)	9 (20.5)
Sex, male	28 (62.2)	35 (79.5)
Site of primary cancer		
Gastric adenocarcinoma	43 (95.6)	42 (95.5)
Gastroesophageal junction	2 (4.4)	2 (4.5)
Metastatic disease	29 (64.4)	25 (56.8)
Tumor histology		
Diffuse	19 (42.2)	17 (38.6)
Intestinal	6 (13.3)	6 (13.6)
Mixed	3 (6.7)	6 (13.6)
Unknown	17 (37.8)	15 (34.1)
ECOG performance status		
0	11 (24.4)	13 (29.5)
1	34 (75.6)	31 (70.5)
Administration of a single dose of mFOLFOX6 during screening	15 (33.3)	13 (29.5)
Prior neoadjuvant or adjuvant therapy for G/GEJC	9 (20.0)	9 (20.5)
FGFR2b expression IHC staining score of 2 + or 3 + in ≥ 10% of tumor cells	29 (64.4)	31 (70.5)

Data are number (%) of patients unless indicated otherwise. The ITT analysis set included all randomized patients

*ECOG* Eastern Cooperative Oncology Group, *FGFR2b* IIIb splice isoform of the fibroblast growth factor receptor 2, *G/GEJC* gastric/gastroesophageal junction cancer, *IHC* immunohistochemistry, *ITT* intention-to-treat, *mFOLFOX6* modified FOLFOX (infusional 5-fluorouracil, leucovorin, and oxaliplatin)

### Progression-free survival

**Fig. 1:**
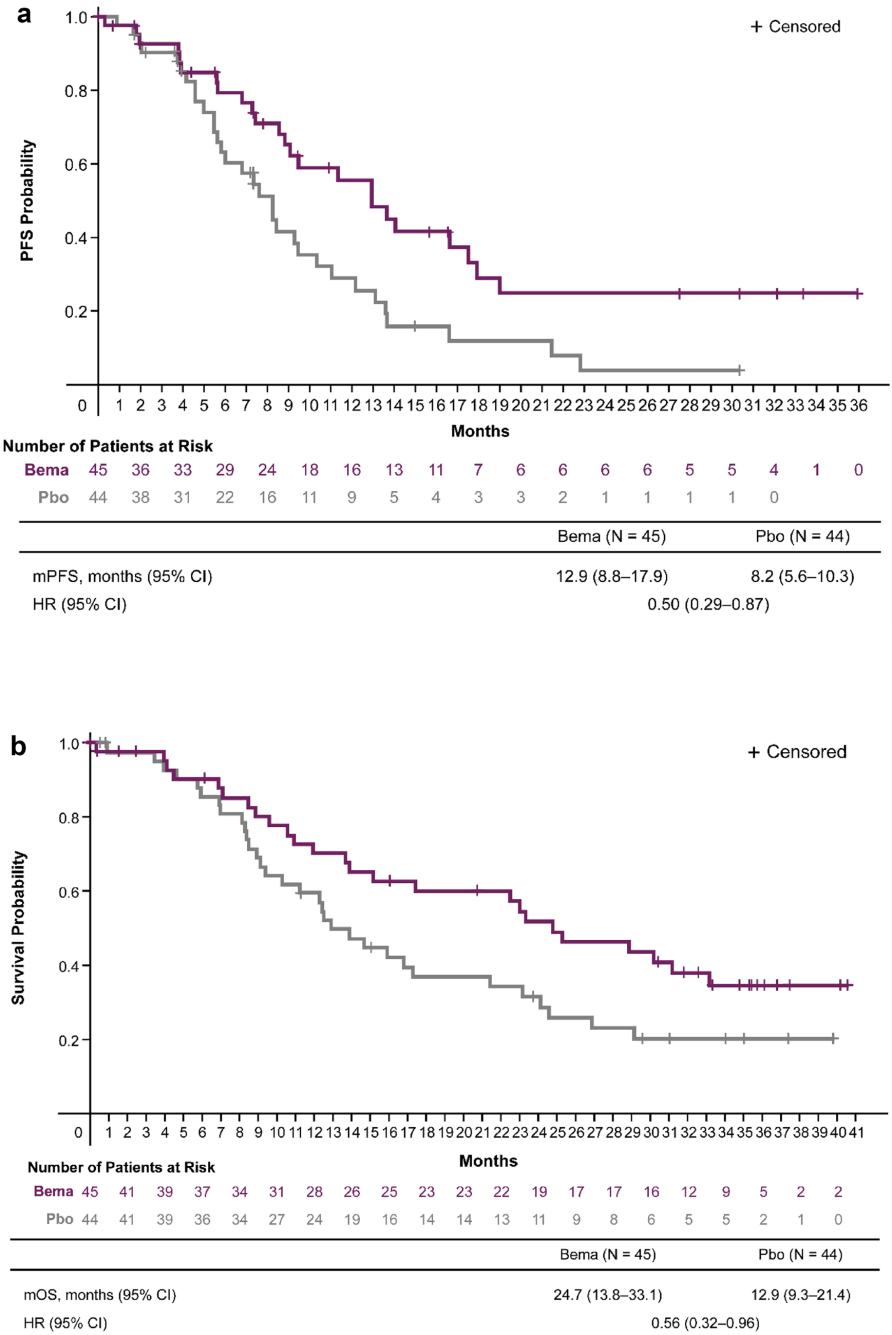
Progression-free survival (**a**) and overall survival (**b**) in the FIGHT East Asian subgroup. *Bema* bemarituzumab plus mFOLFOX6, *CI* confidence interval, *HR* hazard ratio, *mFOLFOX6* modified FOLFOX (infusional 5-fluorouracil, leucovorin, and oxaliplatin), *mOS* median overall survival, *mPFS* median progression-free survival, *OS* overall survival, *Pbo* placebo plus mFOLFOX6, *PFS* progression-free survival. HRs and 95% CIs were calculated using the unstratified Cox proportional hazards model. Vertical bars indicate censoring

At the final analysis data cutoff, median PFS follow-up time was 8.8 months (range, 0–35.9 months). PFS events occurred in 24 (53.3%) patients in the bemarituzumab plus mFOLFOX6 arm and in 32 (72.7%) patients in the placebo plus mFOLFOX6 arm. Median PFS was 12.9 months (95% CI 8.8–17.9 months) with bemarituzumab plus mFOLFOX6 and 8.2 months (95% CI 5.6–10.3 months) with placebo plus mFOLFOX6 (HR 0.50; 95% CI 0.29–0.87), with a 12 month estimated PFS rate of 55.5% (95% CI 37.5–70.2%) and 28.9% (95% CI 14.8–44.7%), respectively (Fig. 1a).

### Overall survival

At the final analysis data cutoff, median OS follow-up time was 14.6 months (range, 0–40.5 months). A total of 25 (55.6%) patients in the bemarituzumab plus mFOLFOX6 arm and 32 (72.7%) patients in the placebo plus mFOLFOX6 arm died. Median OS was 24.7 months (95% CI 13.8–33.1 months) with bemarituzumab plus mFOLFOX6 and 12.9 months (95% CI 9.3–21.4 months) with placebo plus mFOLFOX6 (HR 0.56; 95% CI 0.32–0.96). The estimated OS rate at 12 months was 70.3% with bemarituzumab plus mFOLFOX6 and 59.5% with placebo plus mFOLFOX6; the rates at 24 months were 52.0% and 31.5%, respectively (Fig. 1b).

### Response rate

**Fig. 2:**
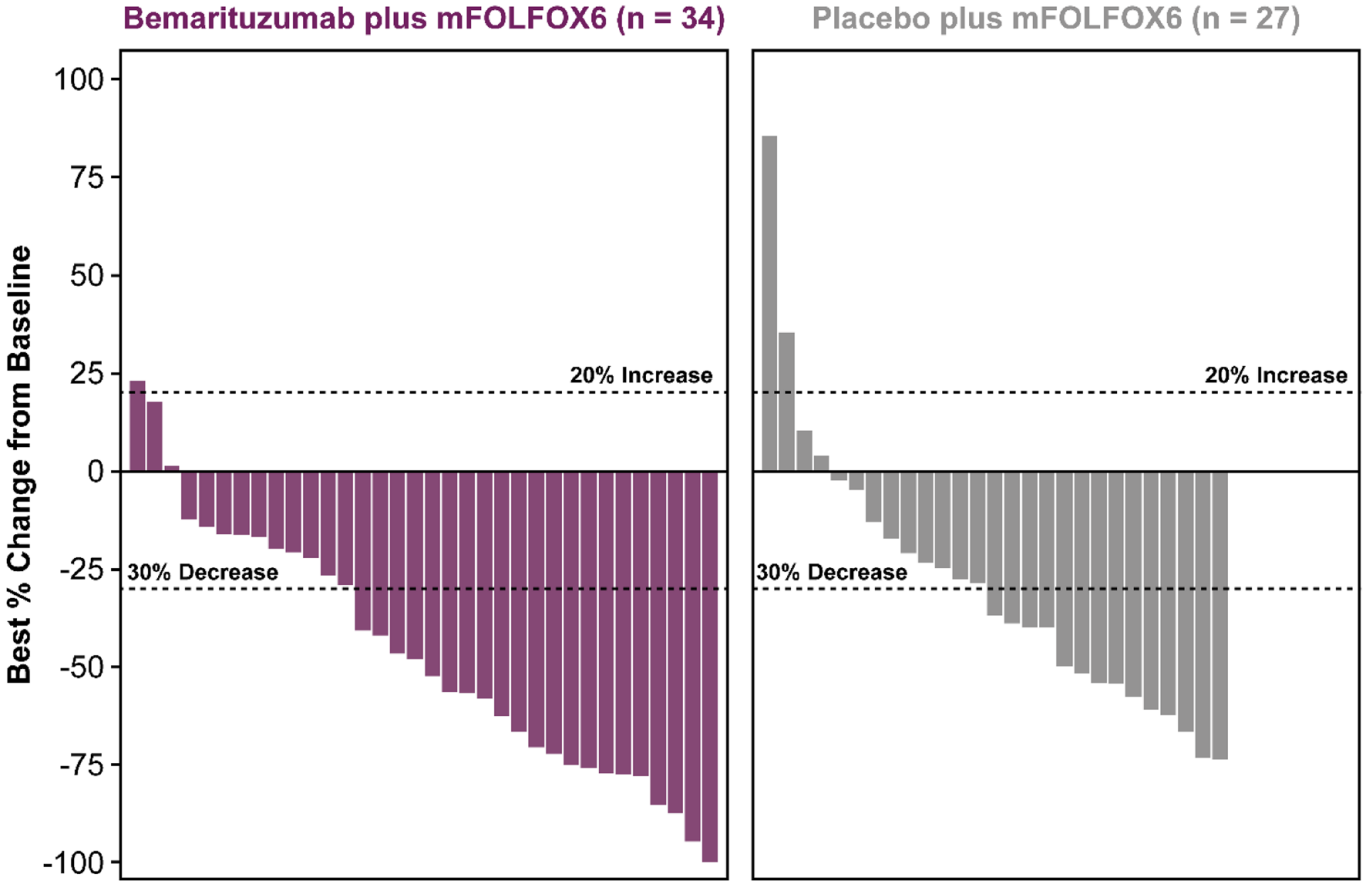
Response by patient in the FIGHT East Asian subgroup. Figure displays data from patients with measurable disease at baseline who were evaluable for evaluation of best overall response

The ORR was 48.9% (95% CI 33.7–64.2%) with bemarituzumab plus mFOLFOX6 and 34.1% (95% CI 20.5–49.9%) with placebo plus mFOLFOX6 (Table 2). CR and PR, respectively, was experienced by 3 (6.7%) and 19 (42.2%) patients in the bemarituzumab plus mFOLFOX6 arm and by 1 (2.3%) and 14 (31.8%) patients in the placebo plus mFOLFOX6 arm. Figure 2 displays the best percentage change in tumor size from baseline by individual patient. The median DOR was 15.6 months (95% CI 9.2–NR) with bemarituzumab plus mFOLFOX6 and 7.6 months (95% CI 3.7–14.8) with placebo plus mFOLFOX6.

**Table 2:** Tumor response in the FIGHT East Asian subgroup

Variable	ITT set		FGFR2b ≥ 10% subgroup	
	Bemarituzumab plus mFOLFOX6 (*N* = 45)	Placebo plus mFOLFOX6 (*N* = 44)	Bemarituzumab plus mFOLFOX6 (*N* = 29)	Placebo plus mFOLFOX6 (*N* = 31)
Measurable disease at baseline	38 (84.4)	30 (68.2)	25 (86.2)	24 (77.4)
Non-measurable disease at baseline	7 (15.6)	14 (31.8)	4 (13.8)	7 (22.6)
Best overall response				
Complete response	3 (6.7)	1 (2.3)	2 (6.9)	0
Partial response	19 (42.2)	14 (31.8)	13 (44.8)	11 (35.5)
Stable disease	17 (37.8)	23 (52.3)	9 (31.0)	14 (45.2)
Progressive disease	2 (4.4)	3 (6.8)	1 (3.4)	3 (9.7)
Not evaluable	4 (8.9)	3 (6.8)	4 (13.8)	3 (9.7)
Objective response rate (ORR)^a^	22 (48.9)	15 (34.1)	15 (51.7)	11 (35.5)
95% CI	(33.7–64.2)	(20.5–49.9)	(32.5–70.6)	(19.2–54.6)
Duration of response^b^				
Number of patients, *n*	22	15	NA	NA
Median (95% CI), months	15.6 (9.2–NR)	7.6 (3.7–14.8)	NA	NA

Data are number (%) of patients unless indicated otherwise. The ITT set included all randomized patients. The FGFR2b ≥ 10% subgroup comprised patients with FGFR2b 2 + /3 + IHC staining in ≥ 10% of tumor cells. Overall response is based on RECIST version 1.1

^a^ORR is computed as the sum of CR and PR according to investigator assessment of tumor lesions per RECIST version 1.1. Two-sided CI based on Clopper-Pearson method

^b^Median duration of response for the FGFR2b ≥ 10% subgroup is not reported because of the low patient numbers in each arm for this analysis

*CI* confidence interval, *CR* complete response, *FGFR2b* IIIb splice isoform of the fibroblast growth factor receptor 2, *IHC* immunohistochemistry, *ITT* intention-to-treat, *mFOLFOX6* modified FOLFOX (infusional 5-fluorouracil, leucovorin, and oxaliplatin), *NA* not applicable, *NR* not reached, *PR* partial response, *RECIST* response evaluation criteria in solid tumors

### Efficacy for patients in FGFR2b ≥ 10% subgroup

Efficacy was assessed among patients in the FGFR2b ≥ 10% subgroup. Median PFS in this subgroup was 17.9 months (95% CI 9.5–NR) with bemarituzumab plus mFOLFOX6 and 7.6 months (95% CI 5.5–9.4) with placebo plus mFOLFOX6 (HR 0.28; 95% CI 0.13–0.57), with estimated 12 month PFS rates of 69.2% (95% CI 45.4–84.2%) and 24.1% (95% CI 9.9–41.6%), respectively (Supplementary Fig. 1a). Median OS was 30.1 months (95% CI 17.3-NR) with bemarituzumab plus mFOLFOX6 and 12.9 months (9.1–16.8) with placebo plus mFOLFOX6 (HR 0.43; 95% CI 0.22–0.86) (Supplementary Fig. 1b). The estimated OS rate at 12 months was 77.4% with bemarituzumab plus mFOLFOX6 and 58.6% with placebo plus mFOLFOX6; the rates at 24 months were 65.4% and 28.2%, respectively. The ORR was 51.7% (95% CI 32.5–70.6%) with bemarituzumab plus mFOLFOX6 and 35.5% (95% CI 19.2–54.6%) with placebo plus mFOLFOX6. CR and PR was experienced by 2 (6.9%) and 13 (44.8%) patients in the bemarituzumab plus mFOLFOX6 arm and by 0 and 11 (35.5%) patients in the placebo plus mFOLFOX6 arm.

### Subsequent therapies

A total of 27 (61%) and 29 (66%) patients in the bemarituzumab plus mFOLFOX6 and placebo plus mFOLFOX6 arms, respectively, received second-line therapy after disease progression (safety analysis set) (Table 3). Taxanes (50%), pyrimidine analogues (27%), ramucirumab (26%), irinotecan (19%), and programmed cell death protein 1/programmed cell death ligand 1 (PD-1/PD-L1) inhibitors (15%) were the most common agents used as subsequent therapy.

**Table 3:** Subsequent anticancer therapy in the FIGHT East Asian subgroup

	Bemarituzumab plus mFOLFOX6 (*N* = 44)	Placebo plus mFOLFOX6 (*N* = 44)
Received at least 1 new anticancer therapy	27 (61.4)	29 (65.9)
Taxanes	21 (47.7)	23 (52.3)
Pyrimidine analogues	12 (27.3)	12 (27.3)
PD-1/PD-L1 inhibitors	8 (18.2)	5 (11.4)
VEGF/VEGFR inhibitor (ramucirumab)	7 (15.9)	16 (36.4)
TOP1 inhibitor (irinotecan/irinotecan HCl)	6 (13.6)	11 (25.0)
VEGFR tyrosine kinase inhibitors	5 (11.4)	3 (6.8)
Platinum compounds	3 (6.8)	4 (9.1)

Data are number (%) of patients. Anticancer therapies were defined as those with an anticancer indication taken after the first dose of study drug. Categories of anticancer therapies taken by at least 5% of randomized subjects are included in the table. The safety set included all randomized patients who received treatment

*HCl* hydrochloride, *mFOLFOX6* modified FOLFOX (infusional 5-fluorouracil, leucovorin, and oxaliplatin), *PD-1/PD-L1* programmed cell death/death ligand 1, *TOP1* topoisomerase 1, *VEGF* vascular endothelial growth factor, *VEGFR* vascular endothelial growth factor receptor

### Safety

All patients in the bemarituzumab plus mFOLFOX6 arm and 43 (97.7%) in the placebo plus mFOLFOX6 arm reported TEAEs; 38 (86.4%) and 34 (77.3%) patients reported grade ≥ 3 TEAEs, respectively (Table 4). A total of 40 (90.9%) and 32 (72.7%) patients in the bemarituzumab plus mFOLFOX6 and placebo plus mFOLFOX6 arms, respectively, reported TEAEs related to bemarituzumab/placebo. A total of 21 (47.7%) and 4 (9.1%) patients in the bemarituzumab plus mFOLFOX6 and placebo plus mFOLFOX6 arms, respectively, reported TEAEs leading to discontinuation of bemarituzumab/placebo. Most TEAEs leading to discontinuation of bemarituzumab were corneal adverse events (17/21 [81%]); no corneal adverse events led to discontinuation of placebo. A total of 7 (15.9%) and 26 (59.1%) patients, respectively, in the bemarituzumab plus mFOLFOX6 arm and 7 (15.9%) and 22 (50.0%) patients in the placebo plus mFOLFOX6 arm reported TEAEs leading to dose reduction and dose delay of bemarituzumab/placebo.

**Table 4:** Incidence of treatment-emergent adverse events in the FIGHT East Asian subgroup

Category	Bemarituzumab plus mFOLFOX6 (*N* = 44)		Placebo plus mFOLFOX6 (*N* = 44)	
Incidence of TEAE	44 (100.0)		43 (97.7)	
Serious TEAEs	14 (31.8)		18 (40.9)	
Grade ≥ 3 TEAEs	38 (86.4)		34 (77.3)	
TEAEs leading to discontinuation of bemarituzumab/placebo	21 (47.7)		4 (9.1)	
Deaths	25 (56.8)		32 (72.7)	
Fatal TEAEs	3 (6.8)		2 (4.5)	
Commonly reported TEAEs with ≥ 25% incidence	Any grade	Grade ≥ 3	Any grade	Grade ≥ 3
Neutrophil count decreased	23 (52.3)	18 (40.9)	23 (52.3)	19 (43.2)
Aspartate aminotransferase increased	22 (50.0)	5 (11.4)	10 (22.7)	1 (2.3)
Nausea	21 (47.7)	0	25 (56.8)	2 (4.5)
Alanine aminotransferase increased	20 (45.5)	2 (4.5)	8 (18.2)	0
Stomatitis	19 (43.2)	5 (11.4)	8 (18.2)	2 (4.5)
Diarrhea	17 (38.6)	2 (4.5)	13 (29.5)	0
Anemia	15 (34.1)	1 (2.3)	18 (40.9)	9 (20.5)
Dry eye	13 (31.8)	1 (2.3)	4 (9.1)	0
Decreased appetite	13 (29.5)	0	19 (43.2)	1 (2.3)
Abdominal pain	12 (27.3)	1 (2.3)	14 (31.8)	2 (4.5)
Peripheral sensory neuropathy	12 (27.3)	5 (11.4)	10 (22.7)	3 (6.8)
White blood cell count decreased	12 (27.3)	5 (11.4)	7 (15.9)	5 (11.4)
Vomiting	11 (25.0)	1 (2.3)	15 (34.1)	0
Platelet count decreased	11 (25.0)	0	9 (20.5)	0
Constipation	9 (20.5)		18 (40.9)	
Fatigue	8 (18.2)	1 (2.3)	11 (25.0)	1 (2.3)
Pyrexia	7 (15.9)	0	13 (29.5)	0

Data are number (%) of patients. The safety set included all randomized patients who received treatment. Adverse events were coded according to MedDRA version 25.0. Severity grades were defined per CTCAE version 5.0. TEAEs began on or after the study drug start date up to 28 days after the last dose of any study drug. Multiple adverse events were counted only once per patient for each PT. PTs are presented by descending order of the total frequencies

*MedDRA* medical dictionary for regulatory activities, *mFOLFOX6* modified FOLFOX (infusional 5-fluorouracil, leucovorin, and oxaliplatin), *PT* preferred term, *TEAE* treatment-emergent adverse event

A total of 14 (31.8%) and 18 (40.9%) patients in the bemarituzumab plus mFOLFOX6 and placebo plus mFOLFOX6 arms, respectively, reported serious TEAEs. A total of 3 (6.8%) and 2 (4.5%) patients in the bemarituzumab plus mFOLFOX6 and placebo plus mFOLFOX6 arms, respectively, reported fatal TEAEs.

A total of 14 (31.8%) patients in the bemarituzumab plus mFOLFOX6 arm and 0 patients in the placebo plus mFOLFOX6 arm reported grade ≥ 3 corneal adverse events; no patients reported serious or grade ≥ 4 corneal adverse events. Corneal adverse events, regardless of grade, were reported by 30 (68.2%) and 6 (13.6%) patients in the bemarituzumab plus mFOLFOX6 and placebo plus mFOLFOX6 arms, respectively; the median time to onset was 17.1 weeks (range, 0.1–38.6) and 12.4 weeks (range, 6.0–29.0), respectively (Table 5). A total of 24 (54.5%) and 1 (2.3%) patient(s) in the bemarituzumab plus mFOLFOX6 and placebo plus mFOLFOX6 arms, respectively, reported grade ≥ 2 corneal adverse events. In the bemarituzumab plus mFOLFOX6 arm, the median time to onset of grade ≥ 2 corneal adverse events was 28.6 weeks (range, 0.1–56.6). A total of 14 patients in the bemarituzumab plus mFOLFOX6 arm experienced resolution or downgrading of grade ≥ 2 corneal adverse events to grade 1, with a median time to resolution/downgrading of 24.5 weeks (range, 2.1–49.7). One patient in the placebo plus mFOLFOX6 arm experienced resolution of a corneal adverse event, with a time to resolution/downgrading of 2 weeks. All corneal adverse events resolved in 15 (34.1%) and 2 (4.5%) patients in the bemarituzumab plus mFOLFOX6 and placebo plus mFOLFOX6 arms, respectively.

**Table 5:** Summary of corneal adverse events

Category	Bemarituzumab plus mFOLFOX6 (*N* = 44)	Placebo plus mFOLFOX6 (*N* = 44)
Any corneal adverse event	30 (68.2)	6 (13.6)
Grade ≥ 3 corneal disorders	14 (31.8)	0
Time to onset of any-grade corneal adverse events		
Number of patients, *n*	30	6
Median (range), weeks	17.1 (0.1–38.6)	12.4 (6.0–29.0)
Time to onset of grade ≥ 2 corneal adverse events		
Number of patients, *n*	24	1
Median (range), weeks	28.6 (0.1–56.6)	NA
Corneal disorders leading to drug discontinuation	17 (38.6)	NA
Commonly reported corneal adverse events		
Dry eye	14 (31.8)	4 (9.1)
Keratitis	7 (15.9)	1 (2.3)
Limbal stem cell deficiency	6 (13.6)	0
Punctate keratitis	4 (9.1)	1 (2.3)
Adverse event resolution category		
All corneal adverse events resolved	15 (34.1)	2 (4.5)
All corneal adverse events resolved or downgraded to grade 1	5 (11.4)	4 (9.1)
Time to resolution of any-grade corneal adverse events		
Number of patients, *n*	17	1
Median (range), weeks	24.1 (1.7–80.1)	NA
Time to resolution or downgrading from grade ≥ 2 to grade 1		
Number of patients, *n*	14	1
Median (range), weeks	24.5 (2.1–49.7)	NA

Data are number (%) of patients unless indicated otherwise. The safety set included all randomized patients who received treatment. Adverse events were coded according to MedDRA version 25.0. Severity grades were defined per CTCAE version 5.0. Time to resolution was calculated among patients with adverse event onset

*CTCAE* common terminology criteria for adverse events, *MedDRA* medical dictionary for regulatory activities, *mFOLFOX6* modified FOLFOX (infusional 5-fluorouracil, leucovorin, and oxaliplatin), *NA* not applicable, *TEAE* treatment-emergent adverse event

## Discussion

East Asian patients treated with bemarituzumab plus mFOLFOX6 experienced a clinically meaningful improvement in median PFS compared with those treated with placebo plus mFOLFOX6 (12.9 vs 8.2 months; HR 0.50; 95% CI 0.29–0.87). Similarly, the treatment benefit in median OS was clinically meaningful with bemarituzumab plus mFOLFOX6 vs placebo plus mFOLFOX6 (24.7 vs 12.9 months; HR 0.56; 95% CI 0.32–0.96). The benefit with bemarituzumab plus mFOLFOX6 was more pronounced in the FGFR2b overexpression ≥ 10% subgroup, with an HR for PFS of 0.28 (95% CI 0.13–0.57) and OS of 0.43 (95% CI 0.22–0.86). Most TEAEs were similar between the two treatment arms except for corneal adverse events, which occurred more frequently with bemarituzumab plus mFOLFOX6 and led to most of the adverse event-related discontinuations in that treatment arm.

As in the overall FIGHT population, patients in the East Asian subgroup experienced robust improvement across efficacy outcomes with bemarituzumab plus mFOLFOX6 [15]. The HR for PFS and OS were 0.72 (95% CI 0.49–1.08) and 0.77 (95% CI 0.52–1.14) in the final analysis of the overall population. ORR was 48.1% (95% CI 36.5–59.7%) with bemarituzumab plus mFOLFOX6 and 33.3% (95% CI 23.1–44.9%) with placebo plus mFOLFOX6 in the overall population, with a median DOR of 11.9 months (95% CI 6.9–17.3) and 7.5 months (95% CI 4.3–13.8), respectively. Treatment benefit was more pronounced in patients with FGFR2b overexpression ≥ 10% in the East Asian subgroup, which was also observed in the overall population (HR for PFS, 0.43 [95% CI 0.26–0.73]; HR for OS, 0.52 [95% CI 0.31–0.85]) [15]. Notably, the HRs for PFS and OS were numerically lower in the East Asian subgroup than those observed in the FIGHT overall population. The study was not sufficiently powered to determine whether this is a meaningful difference. Numerical differences indicating prolonged OS or PFS among Asian patients compared with Western or global trial populations have been observed across trials evaluating immunotherapy for gastric or gastroesophageal cancer [4]. A meta-analysis found statistically significant differences in OS between Asian and Western populations in immunotherapy and first-line gastric cancer clinical trials [4]. Prespecified and post-hoc analyses of PD-1/PD-L1 inhibitors and novel targeted agents in first-line gastric cancer have found that patients enrolled in Asia have numerically improved PFS and OS compared with overall, global study populations [16–21]. The numerical difference in PFS and OS results by region of enrolment in FIGHT and other similar clinical trials may reflect differing treatment patterns in Asia.

A similar proportion of patients reported grade ≥ 3 TEAEs in the overall population (82.9% with bemarituzumab plus mFOLFOX6 and 75.3% with placebo plus mFOLFOX6) and East Asian subgroup [15]. As in the overall population, discontinuation of bemarituzumab were mostly driven by corneal adverse events. This may be due to a protocol requirement that patients in the bemarituzumab were withdrawn if grade ≥ 2 corneal adverse events did not resolve completely or to grade 1. No new safety signals were reported from the East Asian subgroup analysis of the FIGHT trial.

Treatment efficacy of bemarituzumab plus mFOLFOX6 in the FIGHT East Asian subgroup was in line with or improved relative to results of other combinations in front-line gastric cancer, including PD-1/PD-L1 inhibitors and other novel targeted agents. In a systematic meta-analysis, Zhang and colleagues found a significant treatment benefit in Asian patients across clinical trials of immunotherapy (HR, 0.80; 95% CI, 0.65–0.98) [4]. The FIGHT study enrolled patients with untreated, HER-2 non-positive advanced G/GEJC. Studies of novel agents in Asian patients with HER-2 non-positive G/GEJC—the population of the present analysis from the FIGHT trial—demonstrated meaningful treatment benefit. Asian patients with HER-2 non-positive G/GEJC experienced treatment benefit with nivolumab in CheckMate 649 (Chinese patient subgroup analysis) [16, 18] and ATTRACTION-4 (all patients from East Asia) [22], pembrolizumab in KEYNOTE-859 and KEYNOTE-062 (Asian subgroups) [19–21], and zolbetuximab in SPOTLIGHT and GLOW (Asian subgroup) [17]. These results, as well as those of the FIGHT East Asian subgroup, support the use of biomarker-driven agents and immunotherapies in first-line treatment of advanced G/GEJC in East Asian populations.

The results presented here should be considered in the context of strengths and limitations of the analysis. Key strengths of this analysis are that the East Asian subgroup comprised a meaningful proportion of the FIGHT study overall population (reflecting the epidemiology of G/GEJC), and region was a stratification factor in randomization. The key limitations are that this was a post-hoc subgroup analysis, and the sample size was relatively small. Phase 3 global studies of bemarituzumab added to chemotherapy are underway, with enrollment planned across East Asia. Both the FORTITUDE-101 (NCT05052801) [23] and FORTITUDE-102 (NCT05111626) [24] phase 3 trials investigate bemarituzumab plus chemotherapy in previously untreated G/GEJC, and the primary endpoint will be assessed among patients with FGFR2b overexpression in ≥ 10% of tumor cells. The trials will compare bemarituzumab or placebo; all patients will receive mFOLFOX6 in FORTITUDE-101 and nivolumab plus mFOLFOX6/CAPOX in FORTITUDE-102 [23, 24].

## Conclusions

In East Asian patients with advanced or metastatic G/GEJC (*N* = 89), bemarituzumab plus mFOLFOX6 continued to show clinically meaningful outcomes over placebo plus mFOLFOX6 after 24 months of follow-up. As was observed in the global FIGHT final analysis, East Asian patients with FGFR2b overexpression in ≥ 10% of tumor cells by IHC experienced more pronounced treatment benefit. Randomized phase 3 trials are ongoing to confirm the observed clinical benefit of bemarituzumab added to chemotherapy in patients with advanced G/GEJC with FGFR2b overexpression in ≥ 10% of tumor cells by IHC (NCT05111626, NCT05052801) [23, 34]

## Supplementary Information

Below is the link to the electronic supplementary material.

Supplementary file1 (PDF 234 KB)

## Data Availability

Qualified researchers may request data from Amgen clinical studies. Complete details are available at the following: http://www.amgen.com/datasharing.
