# Supplementary material for: Bemarituzumab plus mFOLFOX6 as first-line treatment in East Asian patients with FGFR2b-overexpressing locally advanced or metastatic gastric/gastroesophageal junction cancer: subgroup of FIGHT final analysis
Source: Gastric Cancer. 2024 Jun 11;27(5):1046–57. doi: 10.1007/s10120-024-01516-3 (PMC11335773; doi:10.1007/s10120-024-01516-3)
Supplement: Supplementary file 1 — Supplementary file1 (PDF 234 KB) [file 10120_2024_1516_MOESM1_ESM.pdf]

Supplementary Appendix

Bemarituzumab plus mFOLFOX6 as first-line treatment in East Asian patients with FGFR2b-overexpressing locally advanced or metastatic gastric/gastroesophageal junction cancer: Subgroup of FIGHT final analysis

Yoon-Koo Kang,[1] Shukui Qin,[2] Keun-Wook Lee,[3] Sang Cheul Oh,[4] In-Ho Kim,[5] Jong Gwang Kim,[6] Yong Li,[7] Zhuchen Yan,[8] Jin Li,[9] Li-Yuan Bai,[10] Catherine Chan,[11] Akeem Yusuf,[11] Anita Zahlten-Kümeli,[11] Kate Taylor,[12] Kensei Yamaguchi[13]

[1]Asan Medical Centre, University of Ulsan College of Medicine, Seoul, South Korea; [2]Nanjing Tianyinshan Hospital, The First Affiliated Hospital of China Pharmaceutical University, Nanjing, China; [3]Seoul National University College of Medicine, Seoul National University Bundang Hospital, Seongnam, Gyeonggi-do, South Korea; [4]Department of Internal Medicine, Korea University Guro Hospital, Seoul, South Korea; [5]Department of Oncology, The Catholic University of Korea, Seoul St. Mary's Hospital, Seoul, South Korea; [6]Kyungpook National University Chilgok Hospital, Daegu, South Korea; [7]The Fourth Hospital of Hebei Medical University, Shijiazhuang, Hebei, China; [8]Tianjin Medical University Cancer Institute and Hospital, Tianjin, China; [9]Department of Oncology, Shanghai East Hospital, Shanghai, China; [10]Division of Hematology and Oncology, China Medical University Hospital, and China Medical University, Taichung, Taiwan; [11]Amgen Inc., Thousand Oaks, United States; [12]Amgen Ltd, Uxbridge, United Kingdom; [13]Gastroenterological Chemotherapy Department, The Cancer Institute Hospital of JFCR, Koto-Ku, Tokyo, Japan

Journal: *Gastric Cancer*

Corresponding author: Yoon-Koo Kang, MD, Department of Oncology, Asan Medical Center, University of Ulsan College of Medicine, 88, Olympic-Ro 43-Gil, Songpa-Gu, Seoul, 05505, Korea.

Email: ykkang@amc.seoul.kr

Supplementary Fig. 1. Progression-free survival (a) and overall survival (b) in the FGFR2b overexpression ≥10% subgroup, FIGHT East Asian patients.

a

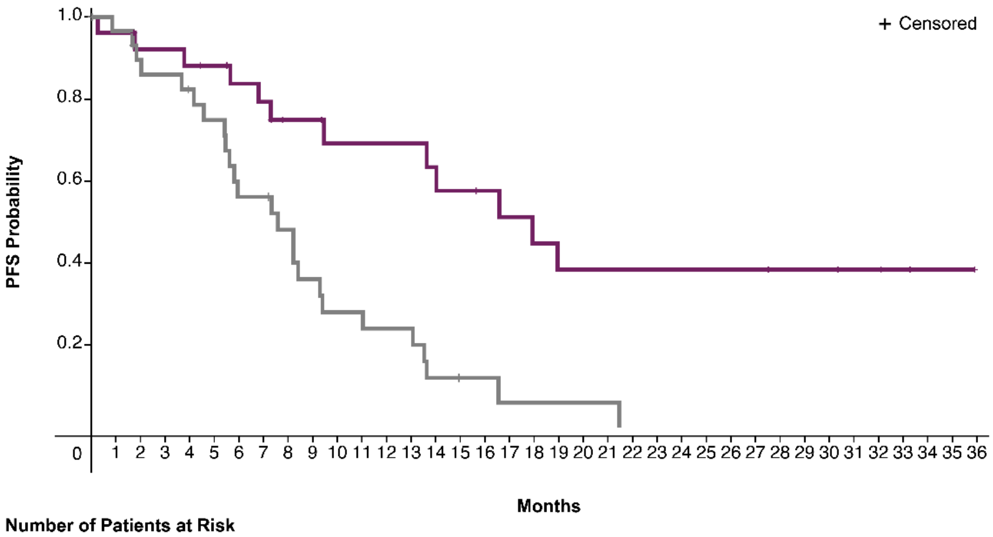

Number of Patients at Risk

Bema	29	23	22	19	15	12	12	11	9	7	6	6	6	6	5	5	4	1	0
Pbo	31	25	22	15	12	7	6	3	2	1	1	0							

	Bema (N = 29)	Pbo (N = 31)
mPFS, months (95% CI)	17.9 (9.5–NR)	7.6 (5.5–9.4)
HR (95% CI)	0.28 (0.13–0.57)	

b

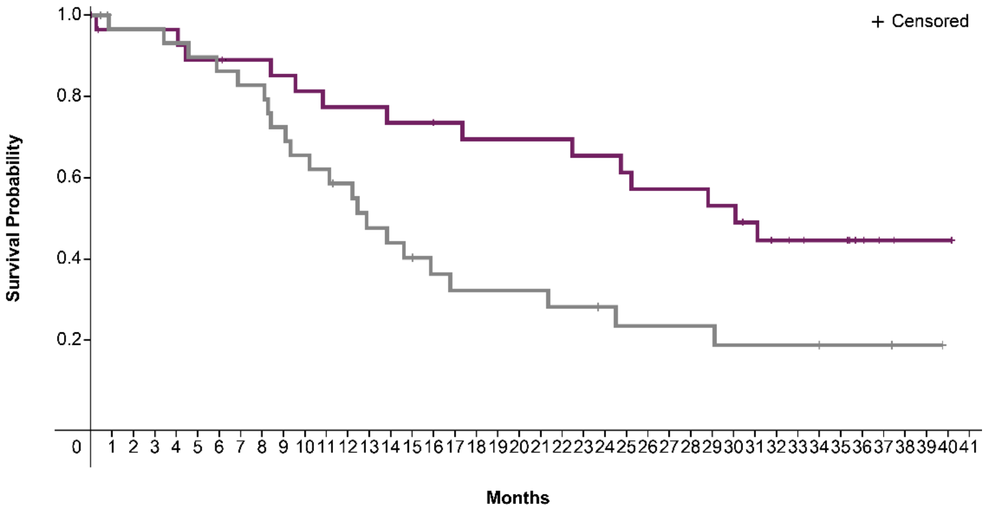

Number of Patients at Risk

Bema	29	26	26	24	23	21	20	19	19	17	17	17	16	14	14	13	9	7	4	1	1
Pbo	31	28	27	25	24	19	16	12	9	8	8	7	6	5	5	4	4	4	2	1	0

	Bema (N = 29)	Pbo (N = 31)
mOS, months (95% CI)	30.1 (17.3–NR)	12.9 (9.1–16.8)
HR (95% CI)	0.43 (0.22–0.86)	

Bema, bemarituzumab plus mFOLFOX6; CI, confidence interval; HR, hazard ratio; mFOLFOX6, modified FOLFOX (infusional 5-fluorouracil, leucovorin, and oxaliplatin); mOS, median overall survival; mPFS, median progression-free survival; OS, overall survival; Pbo, placebo plus mFOLFOX6; PFS, progression-free survival. HRs and 95% CIs were calculated using the unstratified Cox proportional hazards model. Vertical bars indicate censoring.
